# Diversification of chiles (*Capsicum*, Solanaceae) through time and space: New insights from genome-wide RAD-seq data

**DOI:** 10.3389/fgene.2022.1030536

**Published:** 2022-10-18

**Authors:** Carolina Carrizo García, Gloria Estela Barboza, Nahuel Palombo, Hanna Weiss-Schneeweiss

**Affiliations:** ^1^ Multidisciplinary Institute of Plant Biology, Cordoba, Argentina; ^2^ Department of Botany and Biodiversity Research, University of Vienna, Vienna, Austria

**Keywords:** *Capsicum*, wild chiles, phylogeny, range expansion, RAD-seq

## Abstract

*Capsicum* L. encompasses 43 American species, including the five domesticated worldwide consumed sweet and hot chiles. This study presents new, updated and age-calibrated phylogenetic hypothesis on the genus evolution incorporating nearly all currently accepted *Capsicum* species. A new model of spatial diversification of *Capsicum* is proposed based on analyses of several thousands of genome-wide RAD-seq derived SNPs. Maximum likelihood approaches were used to reconstruct phylogenies and to estimate dates of all major splits. Ancestral ranges were inferred and diversification events were modeled in a time frame using a Bayesian approach. Nine clades corresponding to genetically and (mostly) geographically well-defined lineages, which diversified starting around mid-upper Miocene, were recovered with strong support. The Northern and Central Andes were inferred to represent the most likely ancestral range of the genus *Capsicum.* A few early vicariant and dispersal events were estimated to have driven the geographic divergence of the main *Capsicum* clades. Each lineage was inferred to have diversified within a distinct region of South America and expanded geographically to different extent. Extant species diversification was inferred to have begun at the beginning of Pliocene and continued through the Pleistocene. The Central Andes, represented mainly by the territory of present-day Bolivia, were proposed to play a central role in the diversification of lineages comprising domesticated capsicums and their wild allies. The genome-wide approach allowed for high resolution and support of deep phylogenetic nodes providing novel insights into the affinities of major lineages and clades as well as on the geographic expansion of *Capsicum.* This study provides the first dated evolutionary history of the genus encompassing most of the chile species diversity.

## Introduction

The nightshades family (Solanaceae) includes several domesticated species of global relevance, such as potato, tomato, eggplant, tobacco and chiles. Sweet and hot chiles belong to the American genus *Capsicum* L. and are widely used as vegetables and spices around the world. There are five domesticated species in *Capsicum*: *C. annuum* L., *C. chinense* Jacq.*, C. frutescens* L., *C. baccatum* L. and *C. pubescens* Ruiz & Pav., with *C. annuum* being economically most important and including a range of sweet and hot varietals. The species diversity within the genus greatly exceeds that of its domesticated species. Field expeditions across South America have resulted in discovery and description of 13 new *Capsicum* species in recent years, of which six over the last 4 years (Barboza and de Bem Bianchetti, 2005; Nee et al., 2006; Barboza et al., 2011, 2019, 2020a, b, 2022). Thus, the genus *Capsicum* encompasses currently 43 species native to tropical and temperate regions of the Americas, ranging from central Argentina to southern United States (Bosland, 1996; Barboza, 2013; Barboza et al., 2019, 2020a, b, 2022). A detailed and thorough taxonomic treatment has recently been published for *Capsicum* (Barboza et al., 2022) providing a timely opportunity to review the evolutionary history of the genus.

Several hypotheses on the genus evolution have previously been proposed based on single marker DNA sequence analyses using various subsets of *Capsicum* species (Walsh and Hoot, 2001; Jarret and Dang, 2004; Guzmán Díaz et al., 2009; Carrizo García et al., 2016; Barboza et al., 2019, 2020a). Following the latest results, 11 clades have been distinguished within the genus albeit with weak support for some of the branches (cf. Carrizo García et al., 2016; Barboza et al., 2019, 2020a). Specifically, the affinities of *C. flexuosum* Sendtn., *C. longidentatum* Agra & Barboza*, C. coccineum* (Rusby) Hunz., *C. tovarii* Eshbaugh, P.G.Sm. & Nickrent, and *C. pubescens* remained poorly resolved (Carrizo García et al., 2016; Barboza et al., 2019). All DNA-based phylogenetic analyses conducted to date were congruent in the placement of the five domesticated species in the most derived clades of the genus. Similarly, a monophyletic group identified as Andean clade (Carrizo García et al., 2016) has consistently been recovered as the most basal *Capsicum* lineage.

Contrasting hypotheses have been put forward concerning the geographic origin and dispersal of *Capsicum* (McLeod et al., 1982; de Bem Bianchetti, 1996; Moscone et al., 2003; Pozzobon et al., 2006; Carrizo García et al., 2016). The most recent hypothesis based on geographic area modeling suggested the Andean region in western to north-western South America as the ancestral range of origin of the genus, from where the species diversification would have progressively expanded the genus’ range across South America, eventually reaching Central America and southern United States (Carrizo García et al., 2016). This hypothesis was framed within a series of climatic and geological events that strongly influenced the American biota (e.g., Hoorn et al., 2010; Antonelli and Sanmartin, 2011), although the evolutionary history within *Capsicum* was not dated (cf. Carrizo García et al., 2016). No published data concerning the temporal aspect of *Capsicum* diversification and evolution are available in the literature, although general evolutionary timeline has been proposed for the entire Solanaceae family (Särkinen et al., 2013; De-Silva et al., 2017).

Next Generation DNA Sequencing (NGS) technologies generate large amounts of genome-wide DNA sequence data that allow a wide range of analyses for any group of organisms to be performed, regardless of the genetic resources available, and thus enable testing a range of previous hypotheses. One such technology is the Restriction-site-Associated DNA Sequencing (RAD-seq), a reduced-representation sequencing approach that allows scans of the whole genome to characterize overall genetic diversity (Baird et al., 2008). RAD-seq based phylogenetic analyses are used to infer evolutionary histories of genera and families up to ca. 20 million years old (Near et al., 2018), i.e., a period of time spanning the whole *Capsicum* history and beyond (cf. De-Silva et al., 2017). In the current study, the genome-wide RAD-seq methodology was applied to the whole *Capsicum* genus, with more than 80% of species representation, to analyze its diversification and evolution using various phylogenetic and modeling approaches. Specifically, the RAD-seq DNA data were used to reconstruct age-calibrated phylogeny of the whole genus and to address the main events of geographic expansion within a time frame. To this end, the following specific questions were addressed: 1) Is the species diversity of *Capsicum* structured and confined to well-defined clades, with strongly supported inter-clade relationships?, 2) What are the main spatio-temporal patterns of diversification within *Capsicum*? and 3) Can major periods and/or events accompanying the diversification and evolution of the genus be inferred? This study puts special emphasis on the diversity of the wild *Capsicum* species, considering that they have not been subjected to artificial selection by domestication, and therefore they are best suited to unravel the evolutionary history of the chiles.

## Materials and methods

### Plant material, DNA isolation and sequencing

A total of 54 samples of 36 *Capsicum* species representing all currently recognized clades of the genus were included in the analyses [Supplementary Table S1; Carrizo García et al. (2016) and Barboza et al. (2019)]. Only seven species were not be included in the analyses (Supplementary Table S1): *C. benoistii* Hunz. ex Barboza (*insertae sedis*) and six species which belong to the two most speciose clades, Andean [*C. hookerianum* (Miers) Kuntze and *C. regale* Barboza & Bohs] and Atlantic Forest (*C. carassense* Barboza & Bianch., *C. hunzikerianum* Barboza & Bianch., *C. mirum* Barboza and *C. pereirae* Barboza & Bianch.). One *Lycianthes* (Dunal) Hassl. species, the sister genus of *Capsicum*, was used as outgroup (Supplementary Table S1). Samples were collected either in the wild or were obtained from *ex situ* live collections maintained at the HBV (University of Vienna, Austria) and the IMBIV (Argentina). Genomic DNA was isolated using either the DNeasy Plant Mini^®^ kit (Qiagen, United States) or the Invisorb^®^ Spin Plant Mini Kit (Invitek Molecular GmbH, Germany) from leaves dried in silica gel. DNA extracts were purified using the NucleoSpin^®^ gDNA Clean-up kit (Macherey-Nagel GmbH & Co., Germany). DNA was checked for quality by agarose gel electrophoresis and quantified using a Qubit^®^ 3 Fluorometer (Invitrogen, United States). RAD-seq libraries preparation followed the protocol of Paun et al. (2016) with modified settings for DNA fragmentation (i.e., six cycles 90 s off, 60 s on), and using 150 ng for each sample. A two-index combinatorial approach was followed using standard Illumina indexes and inline barcodes. Multiplexed libraries were sequenced (single-end) using an Illumina HiSeq 100bp System (Vienna Biocenter Core Facilities, Austria; https://www.vbcf.ac.at/ngs).

### RAD-seq data filtering

Raw sequence data were de-multiplexed using illumina2bam (https://github.com/gq1/illumina2bam) and process_radtags in Stacks v.2.41 (Catchen et al., 2013) with simultaneous sequence quality filtering (minimum Phred scores 30, allowing a single mismatch in the adapters). Variable SNP loci were filtered and clustered using ipyrad v0.9.45 (Eaton and Overcast, 2020), adjusting only the parameter 21 (the minimum number of samples per locus) and keeping default options in the remaining parameters. Two *de novo* genome-wide datasets were assembled and prepared for different analytical approaches (Supplementary Table S1): 1) extended dataset, comprising all 54 samples of the 36 species studied (Supplementary Data sheet S1), and 2) species dataset, including a single sample per species, except for *C. annuum*, which was represented by its two varieties (Supplementary Data sheet S2). Missing data are expected in RAD-seq data sets and allowing large amounts of missing data would be favorable for phylogenetic analysis (e.g., more parsimony-informative sites, higher overall branch support), particularly when distantly related taxa are involved (Rubin et al., 2012; Huang and Knowles, 2016; Eaton et al., 2017; Crotti et al., 2019). Therefore, test rounds setting percentages of 25, 30, 40, 50, 60, and 70 for the minimum number of samples per locus, specified by the parameter 21 in ipyrad, were performed using both datasets by building maximum likelihood trees. The trees generated were compared and used to define a maximum threshold of missing data for the analyses aiming to generate strongly supported phylogenies. The tree topologies were largely congruent across all main splits in each case, with support values (ultrafast bootstrap and site concordance factors) dropping with the increase of the minimum number of samples per locus (i.e., reducing the amount of missing data). Discrepancies in a few shallow splits (inter-specific affinities) were also observed in the generated trees, with low-moderate support. A minimum of 25% of the samples with data for any given locus was set to filter SNPs for the extended dataset and 30% for the species dataset, based on support values obtained in the test rounds. Output phylip files generated by ipyrad containing a single SNP per locus (∗.usnps.phy files) for both assembled datasets were used for further analyses.

### Phylogenetic inferences

Maximum likelihood (ML) analyses of both datasets were performed using the IQ-Tree software (Nguyen et al., 2015). To address ascertainment bias issues, the ascertainment bias correction (ASC) model was used. Variable sites were filtered out from input datasets producing output alignments which can then be used with the ASC model. The best nucleotide substitution model with lowest BIC score was determined *a priori* using ModelFinder (Kalyaanamoorthy et al., 2017) as implemented in IQ-Tree. The TVM + F model was recovered as the best one for both datasets. Branch supports were calculated with ultrafast bootstrap (UFBoot, 1,000 replicates; Hoang et al., 2018) implemented in IQ-Tree, with UFBoot ≥95% considered to be strong. Site concordance factors (sCF) were also calculated in IQ-Tree (Minh et al., 2020), sampling 100 quartets, as a further measure of branch support. UFBoot/sCF values were provided in that order throughout the text.

### Divergence time analysis

Divergence times of the major splits within *Capsicum* were estimated using the species dataset following a penalized likelihood approach as implemented in the chronos function in the ape package (Paradis and Schliep, 2019) in R (R Core Team, 2020). The ML tree estimated using the species dataset was used as input tree. Two secondary calibration points were applied: 19 million years from the present (myr) for the stem of *Capsicum* and 13.65 myr for the crown node (root) of the genus, following De-Silva et al. (2017). Age estimations used in the current analyses (De-Silva et al., 2017) were revised for the Solanaceae family from earlier fossil-calibrated estimations (Särkinen et al., 2013). The relaxed, correlated, and discrete clock models, with values of the smoothing parameter ʎ from 0 to 5, were tested. The discrete model (i.e., different parts of the tree evolve at different rates) was selected based on the information criterion ΦIC (Paradis, 2013) and the penalized log-Lik values. No differences were found when changing the parameter ʎ, which was therefore set to 0. Divergence times were estimated in million years from the present (myr) as crown ages for the clades.

### Ancestral ranges estimation

Current ranges of all wild *Capsicum* taxa were determined using the verified collection data from Barboza et al. (2022) to define the distributions and areas subsequently used for the analysis. A distribution map was prepared using QGIS 3.16.0-Hannover (QGIS Development Team, 2019); the ESRI Satellite map (ArcGIS/World_Imagery) obtained through the QuickMapServices plug-in (http://nextgis.com/blog/quickmapservices/) was established as basemap layer. Seven areas were defined (Supplementary Figure S1): A. Northern Andes, B. Central Andes, C. Chaco, D. southeastern (SE) South America, E. Amazon basin, F. Central America and southern United States, and G. Galápagos Archipelago. The areas were defined following Thode et al. (2022), with modifications to conform to geological events that have shaped the American landscape from around the time estimated for the origin of *Capsicum* (see Discussion for more details). Unlike in Thode et al. (2022), Northern and Central Andes were here distinguished as separate areas. Three sections have been recognized in the Andes according to their different geological histories, i.e. Northern, Central and Southern (Pérez-Escobar et al., 2022), with *Capsicum* species found in the first two. The Northern and Central Andes are geographically separated by the Huancabamba Depression (Pérez-Escobar et al., 2022), which was marked on the map based on the coordinates specified by Weigend (2004). Ancestral ranges were estimated following the Bayesian binary MCMC method (BBM) in RASP 4.2 (Yu et al., 2015). The ultrametric time-calibrated tree generated with the chronos function using the species dataset was used as input tree. The calibrated tree was saved in parenthetic format and exported to a file with the function write. tree in the ape package. The outgroup taxon as well as most of the domesticated *Capsicum* taxa (*C. annuum* var. *annuum*, *C. frutescens*, *C. pubescens*, *C. baccatum* var. *pendulum* and *C. baccatum* var. *umbilicatum*) were excluded from the analysis as their distribution ranges are artificial. *Capsicum chinense* was the only domesticated species included in the analysis since the data on putative wild forms of this taxon with known geographic location exist (Barboza et al., 2022) and were used as the species distribution for the analysis. The branches of excluded taxa were removed in RASP creating a subset of data (tree and distributions) to be analyzed. Four MCMC chains were run simultaneously for five millions generations, sampling every 1,000 generations, with a 20% burn-in. The model F81 (estimated state frequencies) + G (gamma among-site rate variation) was applied. The maximum number of areas was set to two, given that most taxa included in the analysis were found in one or two areas, rarely in three or more (four out of 34 taxa). Only areas with probability ≥10% were considered in the interpretations of the ancestral ranges estimated; areas with probabilities of ≥90% were considered as unambiguous ranges for the node concerned.

## Results

A total of ≈91 million reads obtained for all the 54 analyzed samples of 36 *Capsicum* species (Supplementary Table S1) were filtered and assembled into the two datasets: 37,944 total loci/SNPs were retained in the extended dataset (54 samples; Supplementary Data Sheet S1) and 42,636 in the species dataset (37 samples; Supplementary Data Sheet S2).

### Phylogenetic analysis of the evolutionary relationships of the genus *Capsicum*

**FIGURE 1:**
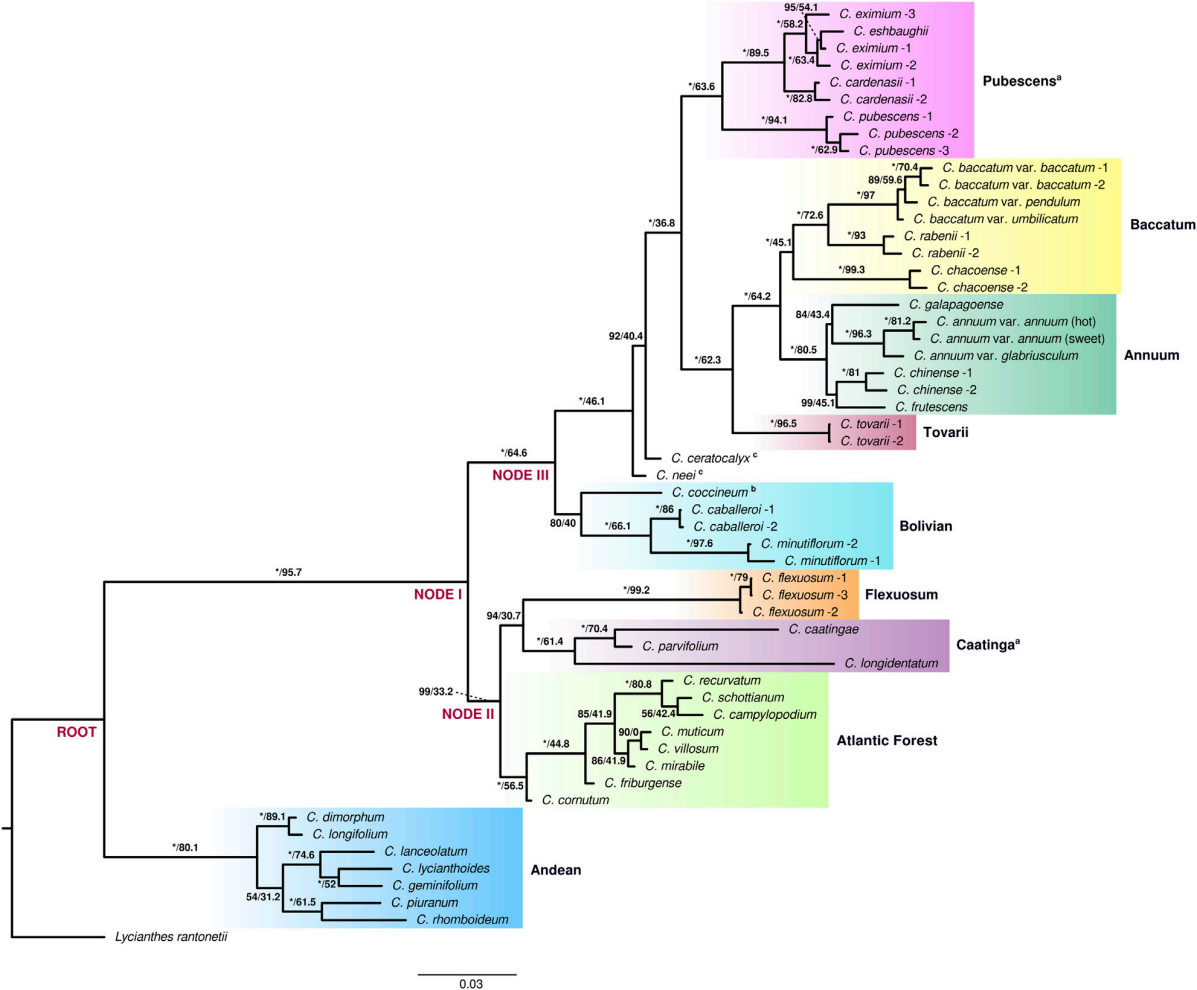
Best-scoring maximum likelihood phylogenetic tree for *Capsicum*. Clades identified are shaded with different colors and labeled; ^a^ clades newly circumscribed, ^b^ provisional placement, ^c^ species not assigned to any clade. Samples identified according to Supplementary Table S1. Support values by branches correspond to ultrafast bootstrap (UFBoot) and site concordant factors. UFBoot = 100% represented by *.

Maximum Likelihood (ML) analysis of both extended and species datasets of the genus *Capsicum* resulted in well resolved and strongly supported tree topologies. Nine clades were recovered consistently in the current analyses: Andean, Atlantic Forest, Flexuosum, Caatinga, Bolivian, Pubescens, Tovarii, Baccatum, and Annuum (Figure 1, Supplementary Figure S2). The topologies of the reconstructed trees were largely congruent (Figure 1, Supplementary Figure S2). Minor differences were observed in the position of only a few species: *C. galapagoense* Hunz. within clade Annuum, the two sister species *C. piuranum* Barboza & S.Leiva and *C. rhomboideum* (Dunal) Kuntze within the Andean clade, and *C. campylopodium* Sendtn., *C. recurvatum* Witasek and *C. schottianum* Sendtn. in the Atlantic Forest clade (Figure 1, Supplementary Figure S2). Two well supported main lineages splitting at the root were recovered within the genus: one lineage that encompassed exclusively the earliest diverging and highly supported Andean clade (100/80.1) with seven species [*C. dimorphum* (Miers) Kuntze, *C. longifolium* Barboza & S.Leiva, *C. piuranum*, *C. rhomboideum*, *C. lanceolatum* (Greenm.) C.V.Morton & Standl. *C. geminifolium* (Dammer) Hunz. and *C. lycianthoides* Bitter], and the second large lineage comprising all of the remaining 29 species, also strongly supported (100/95.7) (Figure 1, Supplementary Figure S2). This second large lineage was again divided into two main sublineages (at node I; Figure 1, Supplementary Figure S2): one encompassing clades Atlantic Forest, Caatinga and Flexuosum (99/33.2) and a second encompassing the remaining five clades: Bolivian, Pubescens, Tovarii, Baccatum and Annuum, with addition of two unassigned species, *C. neei* Barboza & X.Reyes and *C. ceratocalyx* M.Nee (100/64.6). The well-supported Atlantic Forest clade (100/56.5), consisting of eight species (*C. cornutum* (Hiern) Hunz., *C. friburgense* (Bianch. & Barboza), *C. muticum* (Sendtn.) Barboza, *C. mirabile* Mart., *C. campylopodium*, *C. recurvatum*, *C. schottianum* and *C. villosum* Sendtn.; Figure 1, Supplementary Figure S2), split at node II and was sister to two other clades (94/30.7): Caatinga comprising three species (*C. caatingae* Barboza & Agra, *C. parvifolium* Sendtn. and *C. longidentatum*; 100/61.4) and the monotypic Flexuosum clade (*C. flexuosum*; 100/99.2) (Figure 1, Supplementary Figure S2). This sublineage and in particular the position of clade Flexuosum were moderately supported in the phylogeny obtained from the analysis of the species dataset (77/27.9 and 67/29.6, respectively; Supplementary Figure S2). Within the other main sublineage of five clades, the Bolivian clade (80/40), encompassing three species (*C. caballeroi* M.Nee, *C. minutiflorum* (Rusby) Hunz. and *C. coccineum*), diverged at node III (Figure 1, Supplementary Figure S2). Next, two species, *C. neei* and *C. ceratocalyx*, were recovered in isolated positions (Figure 1, Supplementary Figure S2). The positions of *C. coccineum* and *C. ceratocalyx* were mostly moderately supported in the different analyses performed (Figure 1, Supplementary Figure S2). Clades Pubescens, Tovarii, Baccatum, and Annuum were resolved as the most terminal branches of the lineage, and were therefore collectively recognized as the ‘crown group’ of *Capsicum* (100/36.8) (Figure 1, Supplementary Figure S2). These clades encompassed all of the domesticated species and their closest wild relatives. The Annuum (*C. annuum*, *C. chinense*, *C. frutescens* and *C. galapagoense*) and Baccatum (*C. baccatum*, *C. rabenii* Sendtn. and *C. chacoense* Hunz.) clades were recovered as well supported sister groups (100/64.2; Figure 1, Supplementary Figure S2). The monotypic Tovarii clade (*C. tovarii*) was inferred to be sister to the two former ones (100/62.3; Figure 1, Supplementary Figure S2). Clade Pubescens (*C. pubescens*, *C. cardenasii* Heiser & P.G.Sm., *C. eximium* Hunz. and *C. eshbaughii* Barboza; 100/63.6) was recovered as sister group to the assemblage Annuum-Baccatum-Tovarii (Figure 1, Supplementary Figure S2). All the support values provided in this paragraph correspond to the analysis of the extended dataset, unless otherwise indicated.

**FIGURE 2:**
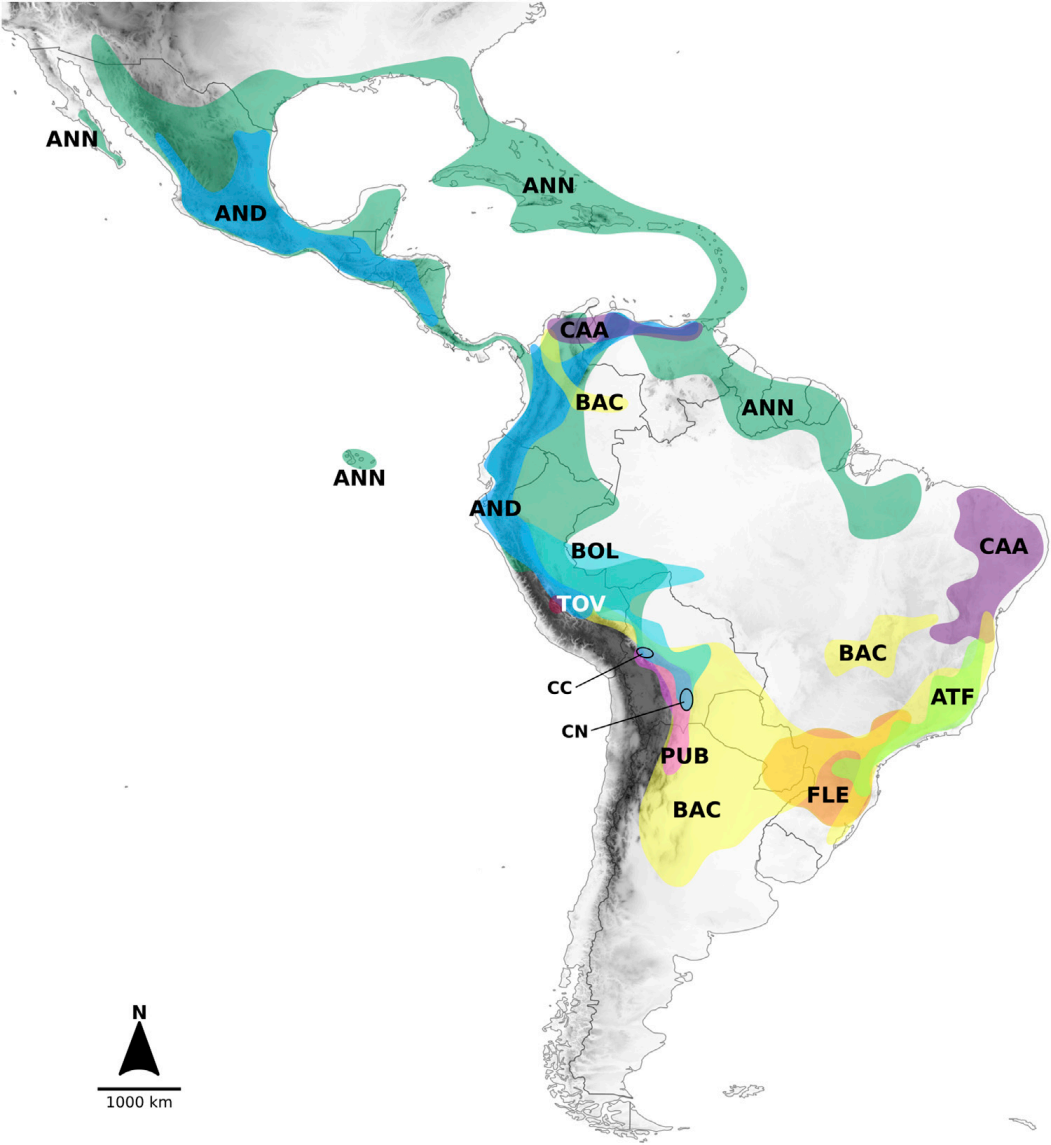
Geographic distribution of the clades recognized in *Capsicum*, including only wild taxa. Distributions outlined according to collection data presented in Barboza et al. (2022). Colors correspond to Figure 1 and Supplementary Figure S2. Legends: AND. Andean clade, ANN. Annuum clade, ATF. Atlantic Forest clade, BAC. Baccatum clade, BOL. Bolivian clade, CAA. Caatinga clade, FLE. Flexuosum clade, PUB. Pubescens clade, TOV. Tovarii clade, species not assigned to any clade = CC. *C. coccineum*, CN. *C. neei*.

The nine clades recovered from the analyses and the lineages in which they segregated were strongly structured geographically (Figure 2). The lineage formed by the Andean clade included species mostly native to the Northern Andes in South America, extending to Central America (Figure 2). The main sublineages splitting within the second large lineage at node I (clades Atlantic Forest, Flexuosum, Caatinga, Bolivian, Pubescens, Tovarii, Baccatum, and Annuum), after the divergence of the Andean clade, also occupied distinct geographical ranges. The species representing clades Caatinga, Flexuosum, and Atlantic Forest (Figure 1, Supplementary Figure S2) that diversified from node II, were mainly distributed in eastern South America (Figure 2) and have thus been referred to as the ‘Eastern’ branch. The remaining sublineage diversifying at node III and comprising clades Bolivian, Pubescens, Tovarii, Baccatum, and Annuum, plus *C. ceratocalyx* and *C. neei*, included species native mostly to the central, western and north-western parts of the South American subcontinent reaching up to Central America and southern United States, with only a few species extending their ranges to eastern territories (e.g., *C. baccatum*, *C. rabenii*) (Figure 2). This branch has thus been referred to as the ‘Western’ branch.

### Divergence times estimation

**FIGURE 3:**
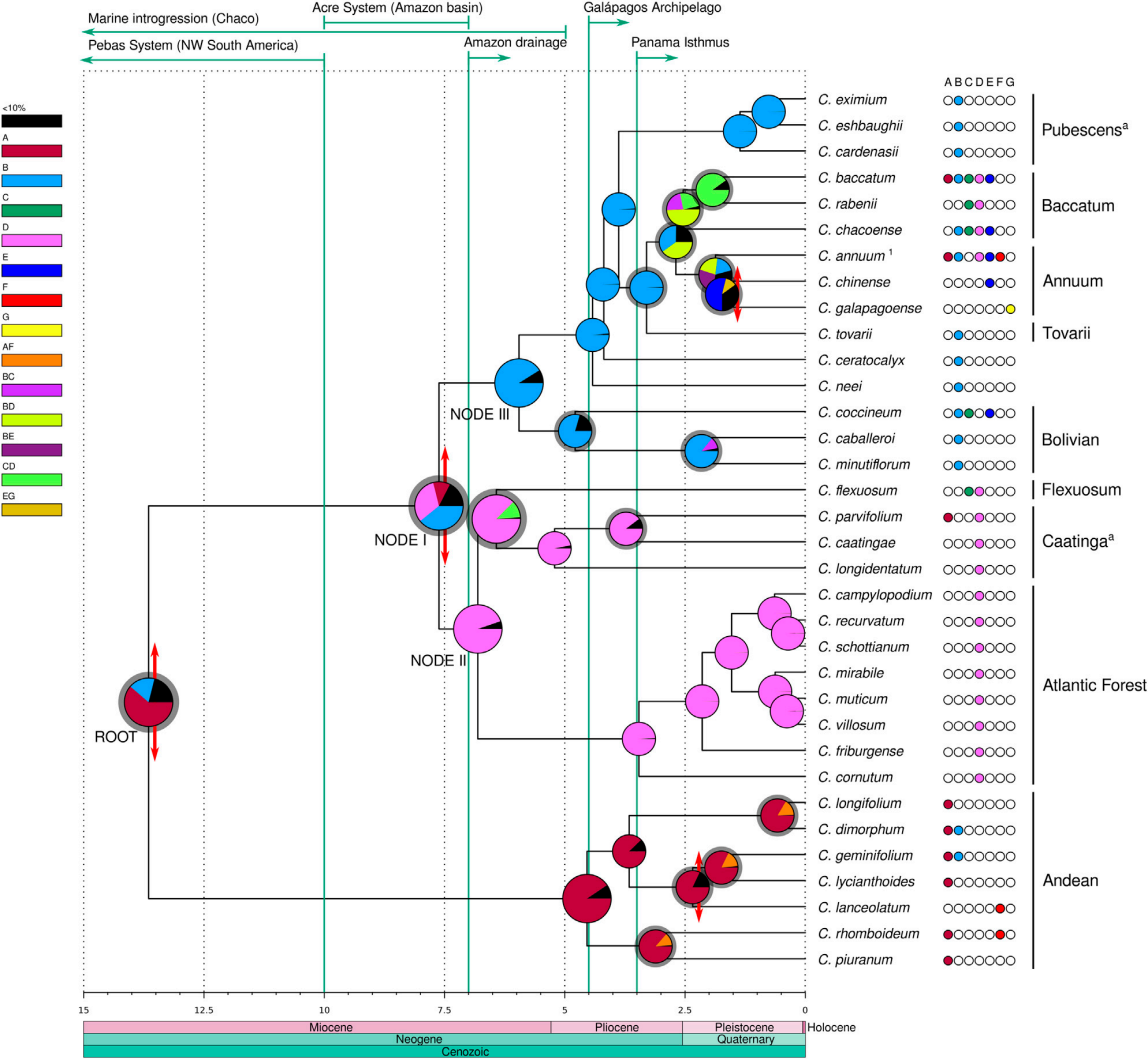
Divergence time and ancestral areas estimated within *Capsicum*. Domesticated taxa with unknown original range are excluded. Recognized clades are labeled; ^a^ clades newly circumscribed. ^1^ Represents *C. annuum* var. *glabriusculum*. Ages expressed in millions years from present. Double-ended red arrows at nodes represent vicariant events, gray shadings at the node pies indicate dispersal events. Major geological events are detailed above, denoted by green arrows/lines. Areas: A. Northern Andes, B. Central Andes, C. Chaco, D. SE South America, E. Amazon basin, F. Central America and southern United States, G. Galápagos Archipelago.

Divergence times were estimated in million years from the present (myr) as crown ages for the clades (Figure 3, Supplementary Figure S3). The Andean clade was inferred to have diverged at the root of the genus at 13.65 myr but the extant species diversification within this clade was estimated to be more recent, starting in the lower Pliocene ca. 4.54 myr. In the second major lineage of *Capsicum*, the ‘Eastern’ (clades Atlantic Forest, Caatinga and Flexuosum) and ‘Western’ (clades Bolivian, Pubescens, Tovarii, Baccatum and Annuum, and *C. ceratocalyx* and *C. neei*) branches at node I diverged from one another 7.62 myr in the upper Miocene. The Atlantic Forest clade diverged 6.81 myr at node II, and the split between the Flexuosum and Caatinga clades was dated to have occurred 6.42 myr, i.e., in the upper Miocene. The Bolivian clade diverged at node III 5.95 myr, *C. neei* 4.42 myr, while the split of *C. ceratocalyx* and the ‘crown group’ was dated at 4.18 myr, all inferred to have taken place at the end of the Miocene through to the lower Pliocene. Within the ‘crown group’, the Pubescens clade diverged 3.88 myr in the mid-Pliocene. *Capsicum tovarii* branch diverged 3.3 myr and clades Annuum and Baccatum split 2.68 myr, during the upper Pliocene. Extant species diversification in all clades was estimated to have mostly taken place between ca. 5 myr (beginning of Pliocene) up to around 0.5–1 myr (mid-upper Pleistocene) (Figure 3, Supplementary Figure S3).

### Ancestral ranges estimation

The ancestral range estimated for the most recent common ancestor (MRCA) of *Capsicum* was inferred most likely to be located in the Andean region (61.6% Northern Andes, 17.6% Central Andes; Figure 3, Supplementary Data Sheet S3). The BBM analysis inferred a total of four vicariant events, 27 dispersals, and no extinctions throughout the diversification history of the genus. An early vicariant event was inferred to have fragmented the ancestral range of *Capsicum* MRCA, after a dispersal event, into the Northern and Central Andes regions, each corresponding to the two main *Capsicum* lineages (Figure 3). Within the Andean clade, the Northern Andes were estimated to represent a strong component across the ancestral ranges, with dispersal events inferred from the Northern Andes towards the Central Andes and Central America (Figure 3). A vicariant event associated with *C. lanceolatum* speciation was inferred to have taken place in Central America in the Pleistocene (Figure 3). The ancestral range at node I was ambiguous, with three putative areas: the Central Andes (39.3%) and SE South America (31.9%) with the highest probabilities, and the Northern Andes (11.1%) with lower probability (Figure 3, Supplementary Data Sheet S3). In the upper Miocene, a vicariant event divided the combined Central Andes/SE South America into the two separate areas, following a range expansion towards SE South America, resulting in the ‘Western’ and ‘Eastern’ branches. SE South America (94.6%) was unambiguously reconstructed as ancestral range at node II and was the dominant ancestral range inferred throughout the ‘Eastern’ branch (Figure 3, Supplementary Data Sheet S3). Dispersal events were inferred from SE South America towards the Chaco (*C. flexuosum*, clade Flexuosum) and the Northern Andes (possibly long-distance dispersal of *C. parvifolium*, clade Caatinga) at different ages (Figure 3). The ancestral range of the MRCA of the ‘Western’ branch at node III was inferred to be the Central Andes (91.4%), and it was also inferred as the ancestral range at all the deep nodes within this branch as well as for the Pubescens clade (99.6%). The Central Andes were recovered as the most likely ancestral range (79.7%) for the Bolivian clade (Figure 3, Supplementary Data Sheet S3). A dispersal event from the ancestral range of the MRCA of clades Tovarii, Baccatum and Annuum was inferred to have occurred towards SE South America. The ancestral range for the MRCA of the Baccatum and Annuum clades, which included the Central Andes/SE South America combined (40.2%) and the Central Andes (35.2%; Figure 3), could not be unambiguously inferred. Dispersals were inferred to have occurred within these areas and towards the Amazon basin. The inferred ancestral range of clade Baccatum was also ambiguous, encompassing three combined areas (Figure 3, Supplementary Data Sheet S3): Central Andes/SE South America (49.4%), Chaco/SE South America (25.4%), and Central Andes/Chaco (22.3%). Dispersal events within ranges as well as range expansions towards the single areas Chaco and SE South America were inferred for this species group. The range of the MRCA of clade Annuum was recovered as ambiguous, including the Central Andes/Amazon basin (27.4%), the Central Andes/SE South America (21.9%), and the Central Andes (18.7%) (Figure 3, Supplementary Data Sheet S3). Several dispersal events from the Central Andes/Amazon basin towards other areas (Northern Andes, SE South America, the Galápagos Archipelago and Central America and southern United States) have been postulated for species of this clade resulting in their range expansions (Figure 3). A vicariant event associated with the origin of *C. galapagoense* in the Galápagos Archipelago was inferred following a (long-distance) range expansion (Figure 3).

Speciation events that have occurred within specific areas were calculated to be five in the Northern Andes, 10 in the Central Andes, two in the Chaco, 13 in SE South America, and one in each of the Amazon basin, the Galápagos Archipelago and Central America and southern United States. Only one speciation event remained ambiguous (i.e., *C. annuum* var. *glabriusculum* (Dunal) Heiser & Pickersgill). Dispersal events were postulated from and within the Northern and Central Andes, the Chaco, SE South America, and the Amazon basin, and towards all seven areas (Supplementary Data Sheet S4). The Central Andes area was the source of the highest number of dispersal events (eight), followed by the Northern Andes and SE South America (five dispersals each; Supplementary Data Sheet S4). The highest numbers of dispersal events within areas were inferred for SE South America (13) and the Central Andes (10; Supplementary Data Sheet S4). Most dispersal events (24 out of 27) were estimated to have occurred through the Pliocene and Pleistocene (Figure 3), mainly concerning the clades Tovarii, Baccatum and Annuum (16 events, multiple at almost all nodes).

## Discussion

This study provides a new robust and updated hypothesis on the diversification and affinities of *Capsicum* species using sequence data representing the snapshots of whole genomes generated through RAD-seq (ca. 37,000–42,000 SNPs). Analyses of these genome-wide datasets allowed to define major phylogenetic lineages and clades (a phylogenetic backbone), date the major splits, and estimate ancestral ranges of clades’ origin along with diversification events with high confidence.

The current results differed to some extent from previous phylogenetic reconstructions of relationships within the genus *Capsicum*, mostly in the recovered relationships between several of the clades and in the position of a few species that have remained weakly supported in previous analyses using single marker DNA sequences (cf. Carrizo García et al., 2016; Barboza et al., 2019, 2020a). The assignment of the individual species to particular clades, however, remained highly congruent with previous studies. Nine well supported clades were identified (Supplementary Table S1), in contrast to 11 clades recognized in the preceding comprehensive genus-wide phylogenetic analysis of *Capsicum* (Carrizo García et al., 2016). Two of previously circumscribed clades, Pubescens and Purple corolla, have now been merged into the enlarged Pubescens clade, named after the most known species of the group. The current results did not support the treatment of *C. pubescens* as an isolated lineage within *Capsicum*, as previously inferred in most phylogenetic reconstructions based on single DNA marker sequence data (Walsh and Hoot, 2001; Carrizo García et al., 2016; 2020; Barboza et al., 2019, 2020a; Guzmán Díaz et al., 2009). Instead, *C. pubescens* was strongly resolved as sister to the former Purple corolla clade species [i.e., *C. eximium*, *C. eshbaughii* and *C. cardenasii* (Carrizo García et al., 2016)]. Such treatment of an extended Pubescens clade is in agreement with the traditional informal placement of its species into the purple flowered group of chiles (Eshbaugh, 1979, 1993; van Zonneveld et al., 2015). The position of *C. longidentatum* has previously been one of the weakest points in the phylogeny of the genus (Carrizo García et al., 2016). This species, recovered earlier as the sole member of the Longidentatum clade (Carrizo García et al., 2016), has now been resolved and placed within the Caatinga clade. This placement was also supported geographically as all three species within this clade are native to the Brazilian Caatinga (Barboza et al., 2011). Only two of all analyzed species, *C. ceratocalyx* and *C. neei*, could not be unequivocally assigned to any of the nine clades since they were recovered in isolated positions.

The recovered phylogenetic relationships contained a strong geographic signal, with most of the clades comprising species that are geographically confined to a certain (eco-)geographic region (Figure 2), with the expected exception of the (widespread) domesticated taxa. All *Capsicum* clades recovered in this study were divided into two major lineages: one encompassing only the earliest splitting Andean clade, diverged at the root ca. 13.65 myr, and the other one including all the remaining species. This is consistent with previous studies which inferred the Andean clade (or any of its species) as basal and markedly divergent, with a set of distinct characters, most remarkably the lack of fruit pungency (Walsh and Hoot, 2001; Carrizo García et al., 2016; Barboza et al., 2019, 2020a). After the split of the Andean clade, the ‘Eastern’ and ‘Western’ branches were distinguished. These encompassed clades centered in the eastern and western regions of South America. The divergence between the ‘Eastern’ and ‘Western’ branches was estimated to have occurred ca. 7.62 myr, almost halfway through the evolutionary history of the genus. The clades Flexuosum and Atlantic Forest were both consistently placed within the “Eastern” branch, which contrasted with their previously inferred, more derived and weakly resolved positions (Carrizo García et al., 2016; Barboza et al., 2019, 2020a). The “Western” branch of *Capsicum* comprised five clades and all domesticated species and their allies (i.e., the “crown group”). From the base of the “Western” branch at node III, five species centered mostly in Bolivian territory, *C. caballeroi*, *C. minutiflorum*, *C. coccineum*, *C. neei* and *C. ceratocalyx*, might have potentially represented rapid diversifying events. *Capsicum neei* and *C. ceratocalyx* formed isolated branches not related to any other individual species/clade, in contrast to previous placements of these taxa within the Bolivian clade (Carrizo García et al., 2016; Barboza et al., 2019). Thus, these two species were ranked here as *insertae sedis* and their relationships will need to be investigated further. The previous placement of *C. coccineum* to the Bolivian clade was supported in the current study, albeit only moderately using the extended dataset. The phylogenetic relationships of *C. coccineum* clearly need further detailed analyses, since its weak affinity to the other Bolivian clade species has been already reported in an earlier study (Barboza et al., 2019). Only a few samples of *C. coccineum* from Bolivian locations have been included in phylogenetic studies so far, thus, much of its genetic diversity might remain unknown (i.e., from Peru and Brazil) given the wide distribution range of this species (Barboza et al., 2022). The “crown group” of *Capsicum* encompassed four clades with all the domesticated species and their closest wild allies. The derived position and internal topology of this group were well resolved and supported. The phylogenetic relationships within the group were largely congruent with previous results based on whole plastome sequence data (D’Agostino et al., 2018; Magdy et al., 2019).

Secondary calibrations alone or in combination with fossil-based calibrations, have been used with confidence in a number of studies to provide dating for major evolutionary events of various plant groups [e.g., Willi et al. (2020) and McDonald-Spicer et al. (2019), respectively]. This study provides first insight into putative timeline of the diversification of the genus *Capsicum*. Many extant Neotropical groups were hypothesized to have begun diversifying during the Miocene/Pliocene (Neogene), reaching the current diversity during the Pleistocene (Quaternary), under the influence of orogenic events and climatic changes occurring during these periods (Rull, 2011; Hughes et al., 2013). Considering the divergence times obtained for *Capsicum*, the timing of the genus diversification was concordant with these hypotheses (see further details below). The extant species diversification within *Capsicum* might have started ca. 5 myr, at the beginning of Pliocene, and continued through the Pleistocene. This trend was consistently observed in all *Capsicum* clades. The three clades encompassing domesticated species, Annuum, Baccatum and Pubescens, might have split at mid Pliocene (clade Pubescens at 3.88 myr, clades Annuum and Baccatum at ca. 2.68 myr). This suggests a long period of divergent evolution of different lineages, of which some species have been subsequently and very recently domesticated by humans [from ca. 10,000 years ago (Perry et al., 2007; Kraft et al., 2014; Dillehay et al., 2017)].

**FIGURE 4:**
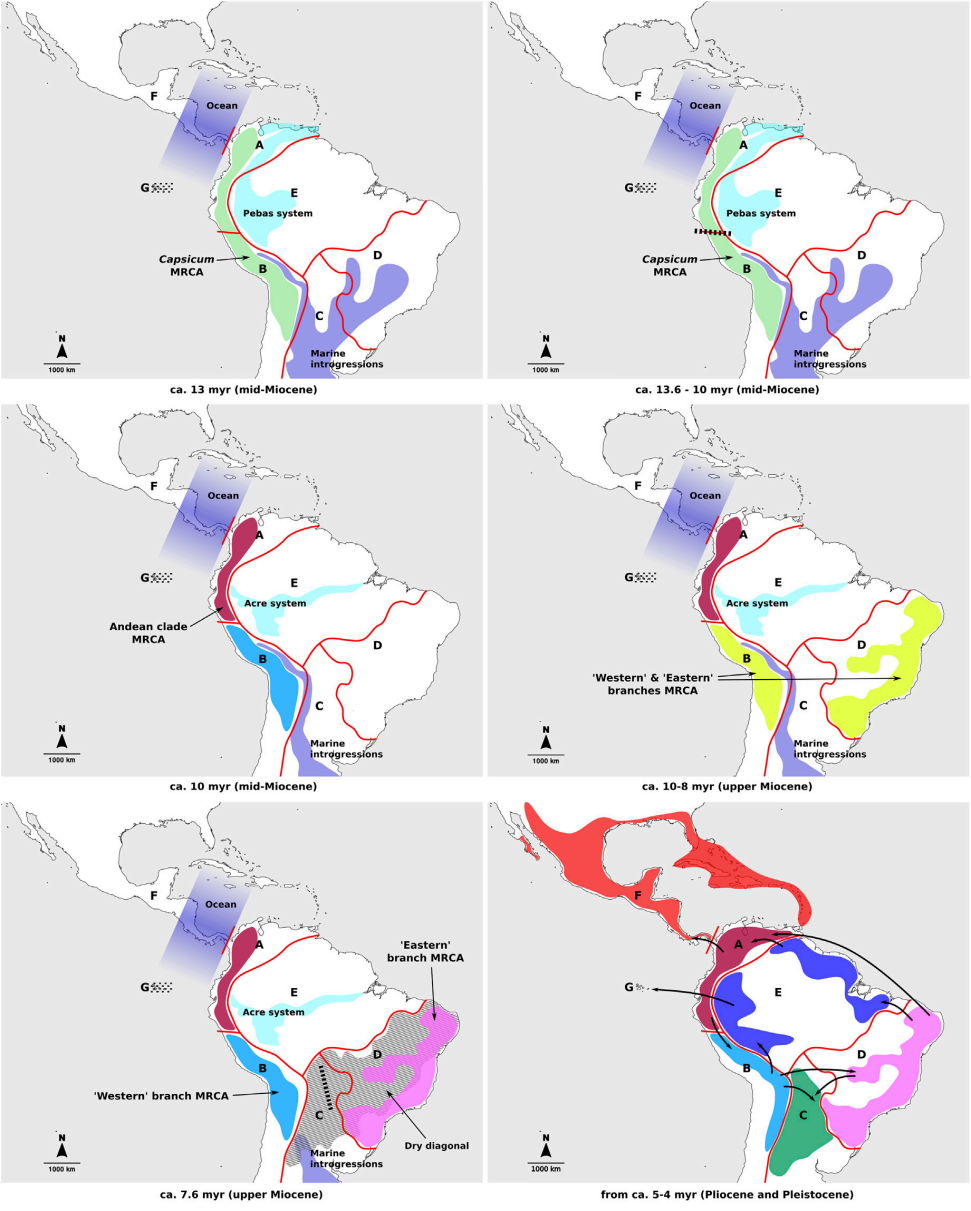
Main events of *Capsicum* spatial diversification through time. Different colors represent current distribution ranges per area. Geographic areas indicated on the figures: A, Northern Andes; B, Central Andes; C, Chaco, D, southeastern South America; E, Amazon basin; F, Central America and southern United States; G, Galápagos Archipelago. Dotted lines indicate islands that are yet to emerge; dashed lines–vicariant events; bold arrows–dispersal events.

The patterns of spatial diversification for *Capsicum* proposed in this study differ from previous hypothesis (Carrizo García et al., 2016), particularly for the earliest stages of the genus evolution, which is consistent with the different tree topologies found at deeper splits. The previous study postulated mainly continuous expansion of ranges between adjacent areas (coded mostly in accordance with political divisions), in clock-wise fashion from the Northern Andes and around South America (Carrizo García et al., 2016). In contrast, the current analysis identified ancient vicariant events, following range expansions, to be decisive in the origin of the main *Capsicum* lineages. These processes resulted in each of the main lineages diversifying separately in different areas of the subcontinent. Late range expansions, linked to most speciation events and a number of dispersals in the Pliocene but mainly in the Pleistocene, would have subsequently generated the extant species distributions (Figure 4). Nonetheless, the relevance of the Northern Andes as part of the putative ancestral range of *Capsicum* MRCA has once again been inferred here.

In the period of time spanning *Capsicum* evolution, extensive geological changes shaped the landscape in South America and likely had a strong impact on the entire American biota (Antonelli et al., 2009; Hoorn et al., 2010; Antonelli and Sanmartín, 2011; Rull, 2011; Pérez-Escobar et al., 2022). In the Miocene, in phase of uplifting of the Andes, major water bodies separated the Andean region from eastern lands in South America (Antonelli et al., 2009; Hoorn et al., 2010; Antonelli and Sanmartín, 2011; Wesselingh and Hoorn, 2011; Pérez-Escobar et al., 2022): the lacustrine Pebas system in the NW and marine introgressions across the center of the subcontinent (Figure 4). South and Central America were then separated by the ocean (Figure 4). During this period, *Capsicum* may have originated in the Andean region and a vicariant event that fragmented the genus ancestral range into Northern and Central Andes may have occurred (Figure 4). Diversification within the early splitting Andean clade continued within the Northern Andes, with subsequent range expansions towards the Central Andes and Central America, the latter estimated to have occurred in the Pleistocene, after the formation of the Isthmus of Panama (Figure 4). The continuous land connection between South and Central America, formed about 3.5 million years ago (Jaramillo, 2018), might have played a role in *Capsicum* dispersals between adjacent South and Central American regions.

The ancestral range of the MRCA of the second major *Capsicum* lineage at node I (ca. 7.62 myr) was reconstructed as ambiguous, with three likely areas to the SE and SW of the Acre system, the latter formed in the upper Miocene (ca. 7–10 myr) in the contemporary Amazon basin (Hoorn et al., 2010) (Figure 4). Long-distance dispersal from the Central Andes towards SE South America could be hypothesized to have originated that range as large parts of the Chaco area might have been submerged in the mid-upper Miocene (Werneck, 2011) (Figure 4). A second vicariant event in the evolution of *Capsicum* would have led to the independent diversification of the “Western” and “Eastern” branches in the Central Andes and SE South America, respectively (Figure 4). A dry diagonal formed by present-day Caatinga, Cerrado and Chaco domains and hypothesized to exist from the Miocene-Pliocene, separated the Atlantic Forest in SE South America from the Amazon Forest in the Amazon basin (Figure 4) (Collevatti et al., 2020). It posed a natural barrier for biotic exchange between both forest areas (Collevatti et al., 2020), although evidence of ancient connections between the two exists (Dias Ledo and Rinaldi Colli, 2017, and references therein). The dry diagonal also separated the Atlantic Forest from the Andes in the west (Figure 4). The distribution of each of the three clades distinguished within the ‘Eastern’ branch (i.e., Atlantic Forest, Caatinga and Flexuosum) corresponded mostly to one of the three domains of SE South America, thus the subsequent diversification within these clades might have been shaped by different local events (e.g., Collevatti et al., 2020), which would deserve more exhaustive sampling and analyses. The third domain included in the dry diagonal, the Chaco, has a different geological history, as it was partially covered by marine waters in part of the Miocene, and was therefore considered as separate in the current study. This area could have acted as a dispersal sink, rather than a source (e.g., dispersal events within the Baccatum clade in the “Western” branch).

The Central Andes were inferred as a major component of the ancestral ranges estimated across the ‘Western’ branch, except for the more derived clades Baccatum and Annuum. This area, represented mainly by the territory of present-day Bolivia, according to the extant species distribution (cf. Barboza et al., 2022), might have played a central role in the diversification leading to the domesticated *Capsicum* species and their wild allies. Clades Baccatum and Annuum encompassed the geographically most widespread species, disregarding the domesticated taxa, which resulted in mostly ambiguous ancestral ranges. Several dispersal events have been suggested to have occurred in these clades in the Pleistocene, resulting in the broad ranges of the species, which expanded beyond the western region of the subcontinent towards Central America and southern United States, the Galápagos Archipelago, the Amazon basin, and SE South America (Figure 4). The occurrence of *C. galapagoense* in the Galápagos Archipelago (4–5 million years old), that would have never had connection to the mainland (Geist et al., 2014; Heads and Grehan, 2021), likely involved long-distance dispersal. The most widely distributed taxon of the genus, *C. annuum* var. *glabriusculum*, is the only one that has colonized southern United States and the Caribbean islands. The detailed evolutionary history of the entire Annuum clade, including domesticated species, certainly requires more exhaustive biogeographical studies.

The Andes harbor the highest number of currently recognized *Capsicum* species (22; cf. Barboza et al., 2022): eight in the Northern Andes, ten in the Central Andes, and four in both. SE South America is the second most *Capsicum*-rich area with 20 species, that can mostly be attributed to the species richness of the Atlantic Forest clade (12 species). The other areas are of lesser significance in terms of species richness (cf. Barboza et al., 2022). This is congruent with the number of speciation events inferred for these areas using the current set of 36 *Capsicum* species.

The present study provides an updated comprehensive phylogenetic hypothesis of relationships within *Capsicum* based on genome-wide DNA data. The relationships were largely very well supported and fully resolved. Nine well-defined clades were recovered, with major splits identified and age-calibrated. The main lineages were distinct and geographically structured. A novel hypothesis on the genus spatio-temporal diversification is presented, in which ancient vicariant and expansion events in mid to upper Miocene have been associated with the origin of the main lineages and clades, while more recent dispersal events in mid Pliocene and Pleistocene could have been responsible for the extant species richness and distribution.

## Data Availability

The datasets supporting the findings of this study are available in the Supplementary Material of this article. The raw data presented in the study are deposited in the NCBI Short Read Archive repository (SRA), BioProject ID PRJNA879205, BioSample accessions SAMN30839235-SAMN30839289.
