# Supplementary material for: Diversification of chiles (Capsicum, Solanaceae) through time and space: New insights from genome-wide RAD-seq data
Source: Front Genet. 2022 Oct 18;13:1030536. doi: 10.3389/fgene.2022.1030536 (PMC9622771; doi:10.3389/fgene.2022.1030536)
Supplement: Supplementary file 3 [file DataSheet3.pdf]

Supplementary Material

1 Supplementary Data

Data S1. RAD-seq SNPs *de novo* alignment of 54 samples from 36 *Capsicum* species (25% minimum number of samples per locus). FASTA file.

Data S2. RAD-seq SNPs *de novo* alignment of single samples from 36 *Capsicum* species (30% minimum number of samples per locus). FASTA file.

Data S3. Ancestral ranges estimated. Detail of probabilities for the ancestral areas of the clades defined and the main splits, after Figure 1. Excel file.

Data S4. Dispersal and speciation events inferred by area in *Capsicum*. Excel file.

2 Supplementary Figures and Tables

2.1 Supplementary Figures

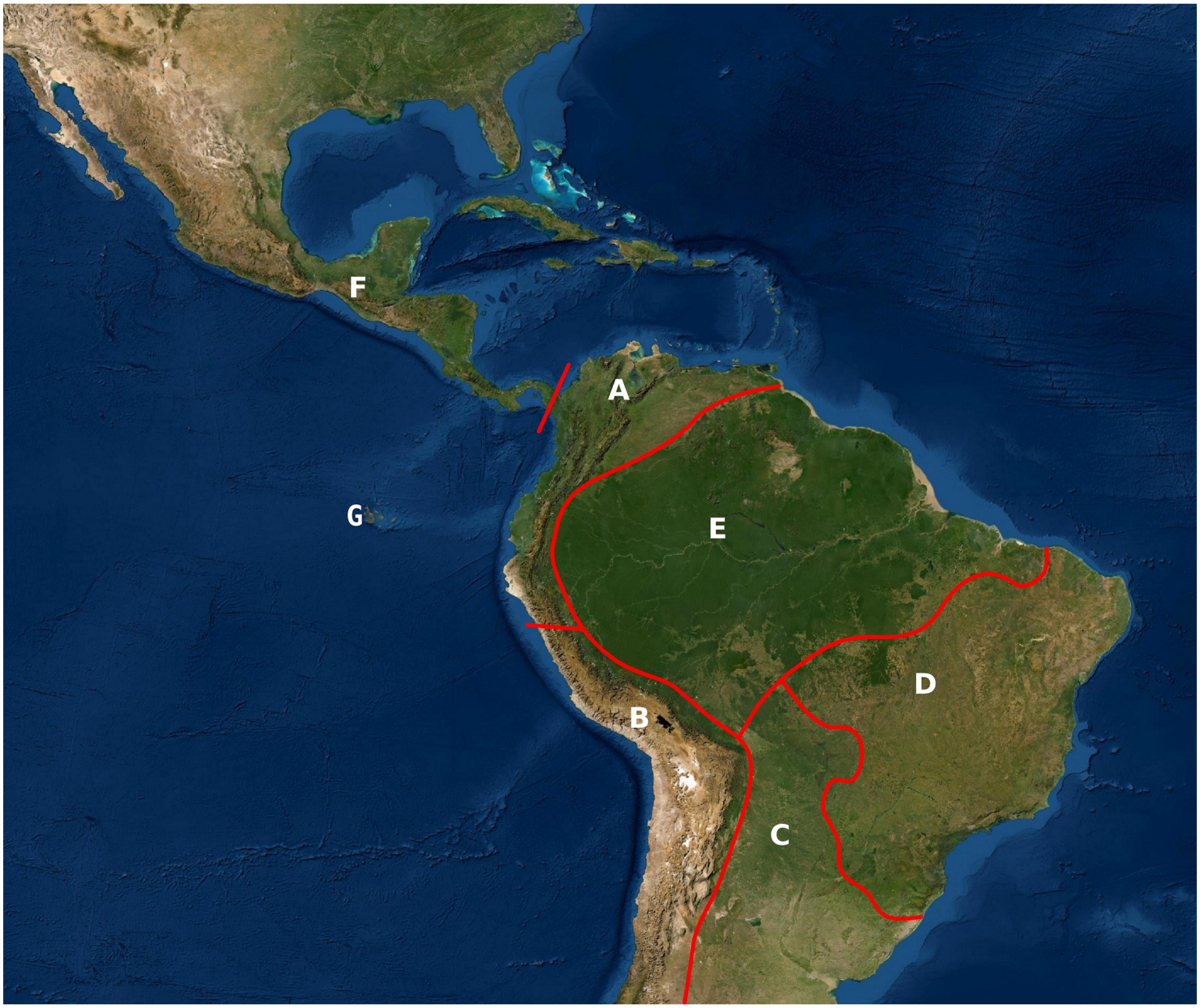

Supplementary Figure S1. Areas delimited to estimate ancestral areas. Current ranges of all wild *Capsicum* taxa were used to outline the genus distribution. Areas: A, Northern Andes; B, Central Andes; C, Chaco, D, southeastern South America; E, Amazon basin; F, Central America and southern United States; G, Galápagos Archipelago.

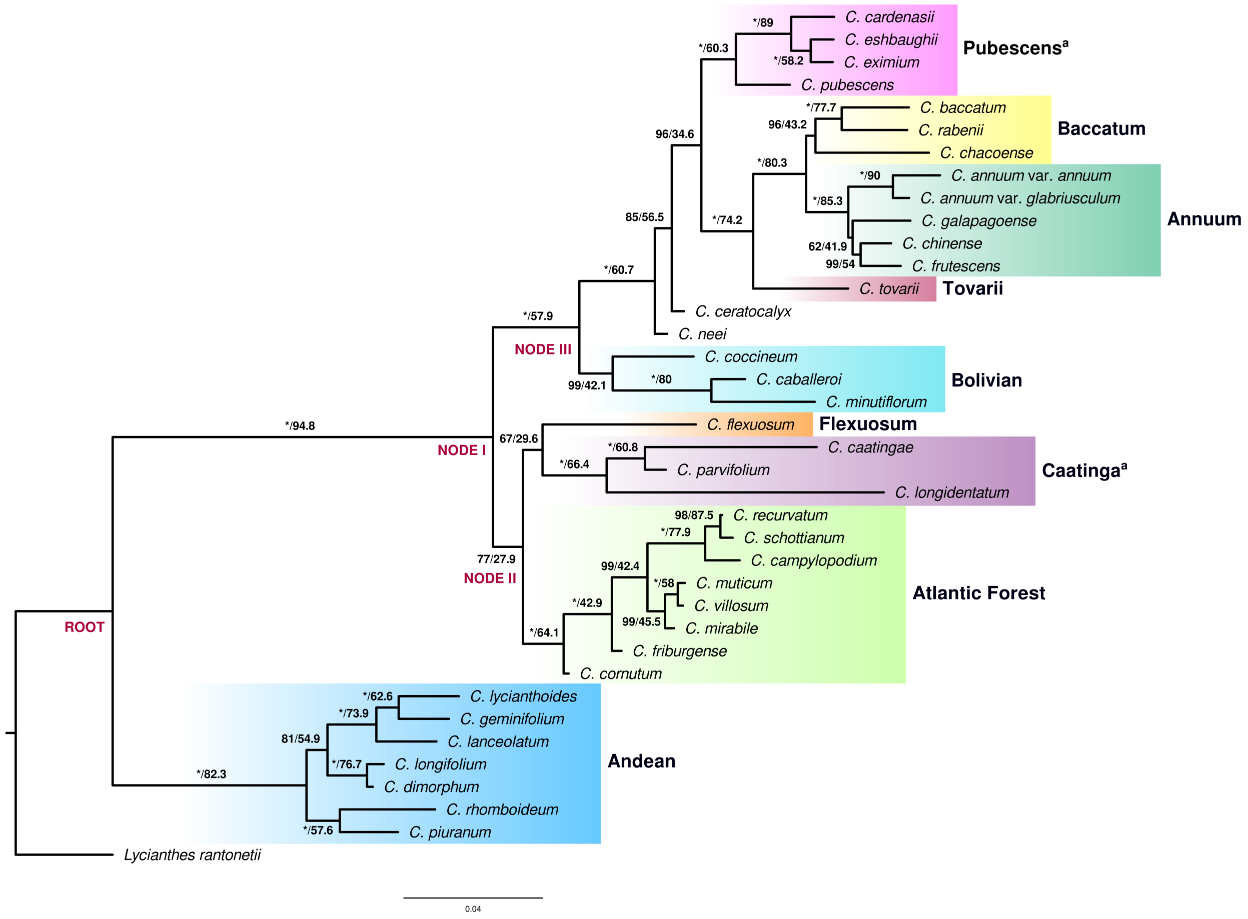

Supplementary Figure S2. Best-scoring maximum likelihood phylogenetic tree for *Capsicum* constructed using a single sample per species. Clades identified are shaded with different colors and labeled; [a] clades newly circumscribed, [b] provisional placement, [c] species not assigned to any clade. Samples identified according to the Table S1. Support values by branches correspond to ultrafast bootstrap (UFBoot) and site concordant factors. UFBoot = 100% represented by *.

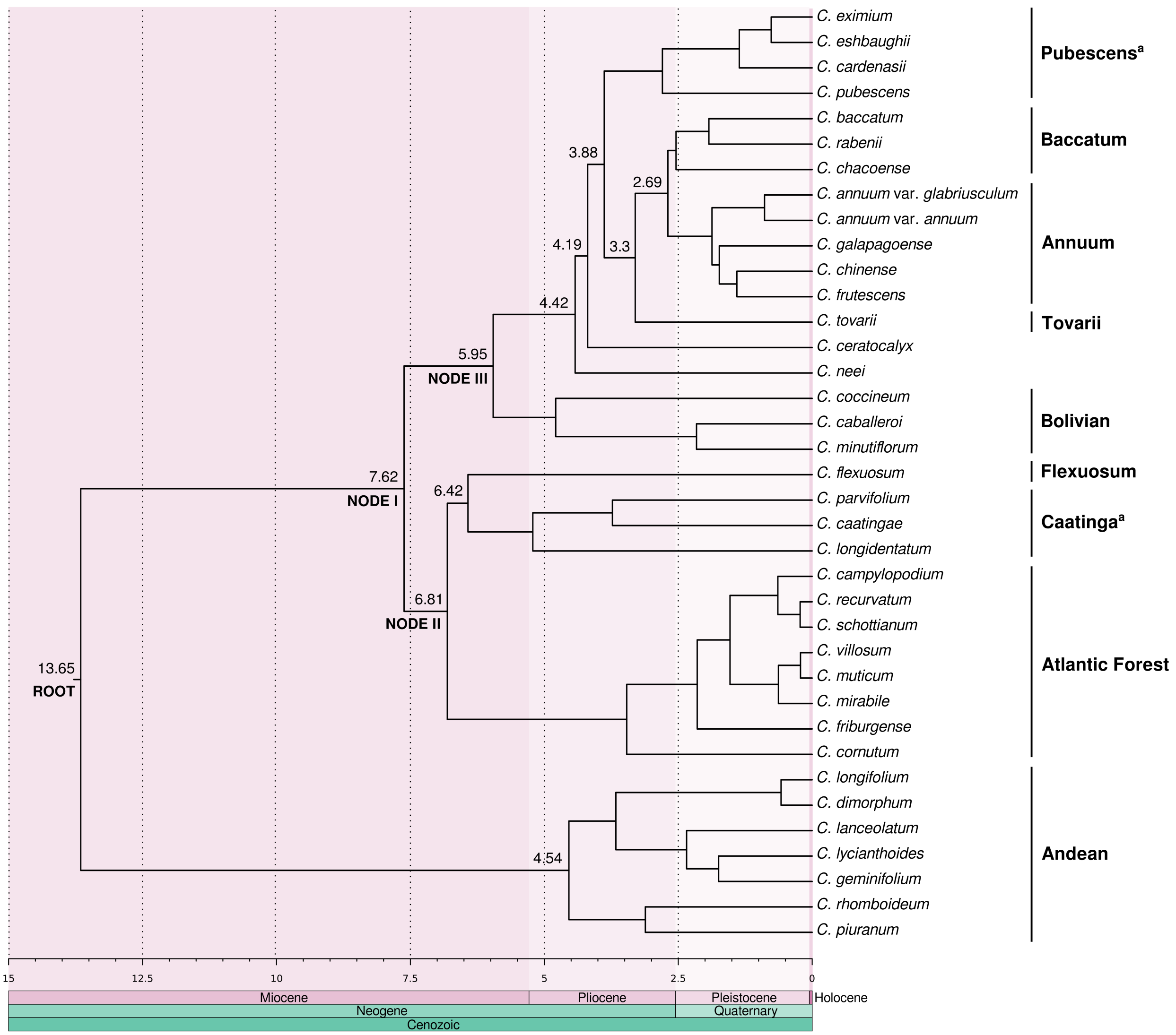

Supplementary Figure S3. Divergence time estimations within *Capsicum*. The chronogram includes all species analyzed, both wild and domesticated. Ages of main splits are specified. Recognized clades are labeled; [a] clades newly circumscribed. Crown ages expressed in millions years from present.

Supplementary Table 1. Clades identified in *Capsicum* and their component species (after Barboza et al., 2022). Materials studied, collection data, and identifications in the datasets. Collection data and identification in the datasets of the materials studied are provided. The outgroup is also included. *Capsicum* species not included in the analyses are highlighted in yellow. * Fruits/seeds obtained in local markets and cultivated *ex situ*.

**Clade** (as currently proposed)	**Species**	**Origin**	**Voucher**	**Extended Dataset – ID**	**Species Dataset**
Andean	*C. dimorphum* (Miers) Kuntze	Colombia: Caquetá	Orejuela 2685	X	X
Andean	*C. geminifolium* (Dammer) Hunz.	Ecuador: Zamora-Chinchipe, E. Científica San Francisco	Orozco 3922	X	X
Andean	*C. hookerianum (*Miers) Kuntze	---	---	---	---
Andean	*C. lanceolatum* (Greenm.) C.V.Morton & Standl.	Cult. Seeds from La Palma Co.	Carrizo García 73	X	X
Andean	*C. longifolium* Barboza & S.Leiva	Ecuador: Zamora-Chinchipe, E. Científica San Francisco	Barboza & Leiva 4851	X	X
Andean	*C. lycianthoides* Bitter	Cult. Seeds from Barboza & Beltrán 85. Colombia: Quindío, Circasia	Barboza & Beltrán 85	X	X
Andean	*C. piuranum* Barboza & S.Leiva	Cult. Seeds from Barboza & Leiva 4841. Peru: Piura, Huancabamba	Barboza & Leiva 4841	X	X
Andean	*C. regale* Barboza & Bohs	---	---	---	---
Andean	*C. rhomboideum* (Dunal) Kuntze	Cult. Seeds from Venezuela: Táchira, San Cristóbal	Carrizo García 97	X	X
Atlantic Forest	*C. campylopodium* Sendtn.	Brazil: Rio de Janeiro, Petrópolis	Barboza et al. 2057	X	X
Atlantic Forest	*C. carassense* Barboza & Bianch.	---	---	---	---
Atlantic Forest	*C. cornutum* (Hiern) Hunz.	Brazil: São Paulo, Paranapiacaba	Barboza & Cosa 2517	X	X
Atlantic Forest	*C. friburgense* Bianch. & Barboza	Brazil: Rio de Janeiro, Nova Friburgo	Barboza et al. 2048	X	X

**Clade**<br>(as currently proposed)	**Species**	**Origin**	**Voucher**	**Extended Dataset – ID**	**Species Dataset**
Atlantic Forest	*C. hunzikerianum* Barboza & Bianch.	---	---	---	---
Atlantic Forest	*C. mirabile* Mart.	Brazil: São Paulo, Campos do Jordao	Barboza & Carrizo García 3632	X	X
Atlantic Forest	*C. mirum* Barboza	---	---	---	---
Atlantic Forest	*C. muticum* (Sendtn.) Barboza	Brazil: Rio de Janeiro, Petrópolis	Barboza & Carrizo García 3946	X	X
Atlantic Forest	*C. pereirae* Barboza & Bianch.	---	---	---	---
Atlantic Forest	*C. recurvatum* Witasek	Cult. Seeds from Barboza 1632. Brazil.	Barboza 1632	X	X
Atlantic Forest	*C. schottianum* Sendtn.	Cult. Seeds from Barboza 3635. Brazil: São Paulo, Paranapiacaba	Barboza 3635	X	X
Atlantic Forest	*C. villosum* Sendtn.	Brazil: Rio de Janeiro	Barboza et al. 1653	X	X
Flexuosum	*C. flexuosum* Sendtn.	Cult. Seeds from Barboza 1034. Argentina: Misiones, Candelaria	Barboza 1034	X – 1	X
Flexuosum	*C. flexuosum*	Cult. Seeds from unknown Brazilian origin, retrieved from an old collection.	Carrizo García 84	X – 2	
Flexuosum	*C. flexuosum*	Cult. Seeds from Daviña & Hofni 599. Argentina: Misiones, Candelaria	Daviña & Hofni 599	X – 3	
Caatinga	*C. caatingae* Barboza & Agra	Brazil: Paraíba, Morro do Quixaba	Agra & Barboza 7085	X	X
Caatinga	*C. longidentatum* Agra & Barboza	Brazil: Bahia, Itatim	Agra et al. 7083	X	X
Caatinga	*C. parvifolium* Sendtn.	Brazil: Paraíba, Matureia	Agra & Barboza 7075	X	X
Bolivian	*C. caballeroi* M.Nee	Bolivia: Santa Cruz, El Empalme	Barboza 4907	X – 1	

**Clade** (as currently proposed)	**Species**	**Origin**	**Voucher**	**Extended Dataset – ID**	**Species Dataset**
Bolivian	*C. caballeroi*	Bolivia: Santa Cruz, El Empalme	Barboza 3655	X – 2	X
Bolivian	*C. coccineum* (Rusby) Hunz.	Bolivia: Santa Cruz, La Chonta	Barboza 4921	X	X
Bolivian	*C. minutiflorum* (Rusby) Hunz.	Bolivia: Tarija, Bermejo	Carrizo García & Fernández 64	X – 1	X
Bolivian	*C. minutiflorum*	Bolivia: Santa Cruz, Tartarenda	Carrizo García & Fernández 61	X – 2	
Pubescens	*C. cardenasii* Heiser & P.G.Sm.	Cult. Bolivia: La Paz *	Carrizo García 76	X – 1	
Pubescens	*C. cardenasii*	Cult. Bolivia: La Paz, Sorata *	IMBIV, live collection	X – 2	X
Pubescens	*C. eshbaughii* Barboza	Bolivia.: Santa Cruz, Samaipata	Carrizo García & Fernández 67	X	X
Pubescens	*C. eximium* Hunz.	Bolivia: Chuquisaca, La Palizada	Carrizo García et al. 31	X – 1	
Pubescens	*C. eximium*	Bolivia: Santa Cruz, Vallegrande	Carrizo García et al. 39	X – 2	
Pubescens	*C. eximium*	Argentina: Jujuy, El Fuerte	Carrizo García & Fernández 53	X – 3	X
Pubescens	*C. pubescens* Ruiz & Pav.	Cult. Seeds from Barboza et al. 3658	Carrizo García 75	X – 1	X
Pubescens	*C. pubescens*	Cult. Mexico: Coyoacán *	IMBIV, live collection	X – 2	
Pubescens	*C. pubescens*	Cult. Peru: Piura, Piura *	IMBIV, live collection	X – 3	
Tovarii	*C. tovarii* Eshbaugh, P.G.Sm. & Nickrent	Cult. Seeds from CGN, PI 606708/NMCA 90008 -Ind. 1	Carrizo García 74	X – 1	X
Tovarii	*C. tovarii*	Cult. Seeds from CGN, PI 606708/NMCA 90008 -Ind. 2	Carrizo García 74	X – 2	

**Clade** (as currently proposed)	**Species**	**Origin**	**Voucher**	**Extended Dataset – ID**	**Species Dataset**
Baccatum	*C. baccatum* L. var. *baccatum*	Argentina: Jujuy, Santa Silvina	Carrizo García & Fernández 47	X – 1	
Baccatum	*C. baccatum* var. *baccatum*	Argentina. Jujuy, San Francisco	Palombo 1	X – 2	X
Baccatum	*C. baccatum* var. *pendulum* (Willd.) Eshbaugh	Cult. Seeds from Barboza 3642	Carrizo García 96	X	
Baccatum	*C. baccatum* var. *umbilicatum* (Vell.) Hunz. & Barboza	Cult. Argentina: Cordoba *	HBV, live collection	X	
Baccatum	*C. chacoense* Hunz.	Argentina: Tucumán, Vipos	Carrizo García & Fernández 83	X – 1	
Baccatum	*C. chacoense*	Argentina: Salta, Güemes	Palombo 16	X – 2	X
Baccatum	*C. rabenii* Sendtn.	Cult. Seeds from CGN 22795	Carrizo García 86	X – 1	
Baccatum	*C. rabenii*	Cult. Seeds from Barboza 1656. Brazil: São Paulo, Bananal	Carrizo García 87	X – 2	X
Annuum	*C. annuum* L. var. *annuum*	Origin unknown * (“Lippenstift”)	HBV, live collection	X – sweet	X
Annuum	*C. annuum* var. *annuum*	Origin unknown * (“Apfelpaprika”)	HBV, live collection	X – hot	
Annuum	*C. annuum* var. *glabriusculum* (Dunal) Heiser & Pickersgill	Cult. Seeds from NMCA 10983 (USA)	Carrizo García 102	X	X
Annuum	*C. chinense* Jacq.	Origin unknown * (“Gänseschnabel”)	HBV, live collection	X – 1	X
Annuum	*C. chinense*	Origin unknown * (“Trinidad Moruga Yellow”)	HBV, live collection	X – 2	
Annuum	*C. frutescens* L.	Cult. Seeds from Barboza 2064.	HBV, live collection	X	X
Annuum	*C. galapagoense* Hunz.	Cult. Seeds obtained from CGN 22208	Carrizo García 99	X	X

**Clade** (as currently proposed)	**Species**	**Origin**	**Voucher**	**Extended Dataset – ID**	**Species Dataset**
Insertae sedis	*C. benoistii* Hunz. ex Barboza	---	---	---	---
Insertae sedis	*C. ceratocalyx* M.Nee	Bolivia: La Paz, Nor Yungas, Florida	Beck 33135	X	X
Insertae sedis	*C. neei* Barboza & X.Reyes	Bolivia: Chuquisaca, Hernando Siles, Monteagudo	Barboza 4927	X	X
**Outgroup**	*Lycianthes rantonnetii* (Carrière) Bitter	Argentina: Córdoba, cult. (origin unknown)	Carrizo García 29	X	X
